# Percutaneous Biliary Neo-Anastomosis of Inadvertently Operatively Excluded Right Posterior Bile Ducts: A Durable and Highly Successful Procedure

**DOI:** 10.1007/s00270-025-03977-0

**Published:** 2025-02-10

**Authors:** D. Nakhostin, Henrik Petrowsky, G. Puippe, A. Kobe, T. Pfammatter

**Affiliations:** 1 Institute of Diagnostic and Interventional Radiology, University Hospital Zurich, Zurich, Switzerland; 2 Clinic of Visceral and Transplantation Surgery, University Hospital Zurich, Zurich, Switzerland

**Keywords:** Biliary anastomosis, Bile leakage, Percutaneous transhepatic biliary drainage

## Abstract

**Purpose:**

To evaluate the feasibility and outcome of percutaneous biliary neo-anastomosis (PBNA) by means of fluoroscopy-guided enteral/biliary puncture, followed by temporary percutaneous transhepatic biliary drainage (PTBD).

**Materials and Methods:**

Four patients (25% female, mean age 56 years) were referred for PBNA (bilio-entero-neo-anastomosis, *n* = 3; bilio-biliary-neo-anastomosis, *n* = 1) between 2007 and 2021. They presented with right posterior bile duct exclusion and bile leakage after major liver or pancreatic surgery, or hemiliver transplant. After puncture of the excluded/leaking bile duct, neo-anastomosis was performed under fluoroscopic guidance using the back end of a 0.018″ guidewire (*n* = 3) or a vascular reentry device (*n* = 1). PTBDs were inserted to assure PBNA healing.

**Results:**

Technical success rate was 100%. No complications occurred. All PTBDs were removed (median = 65 days). During a median follow-up of 2.8 years, two patients died due to unrelated causes. No subsequent bile leakages or re-occlusions were observed.

**Conclusion:**

In conclusion, PBNA is feasible and safe, and offers long-term biliary patency even in liver-transplanted patients.

## Introduction

The anatomical variant consisting of a low right posterior sectoral duct insertion into the hepatic or even the cystic duct places it at risk to be injured at laparoscopic cholecystectomy [1, 2]. Hepaticojejunostomy has been considered as the mainstay of treatment as these injuries became more frequent with the advent of laparoscopic cholecystectomy. Currently, injuries of the right posterior sector duct at laparoscopic cholecystectomy may well be managed non-operatively by endoscopic or interventional radiological techniques [2, 3]. However, early re-laparotomy may be hazardous after major open hepato-biliary or pancreatic surgery and endoscopic management consisting of sphincterotomy and insertion of nasobiliary catheters or stent (-grafts) may fail if continuity of a transected duct cannot be established.

We present four symptomatic consecutive patients with inadvertently excluded right posterior sectoral bile ducts at hepato-biliary or pancreatic (HPB) surgery (Type C injuries according to the Strasberg classification) [4]. As these patients were unfit for early redo bilio-enteric anastomosis (*n* = 4) and endoscopic treatment had failed (*n* = 1), percutaneous fluoroscopy-guided biliary neo-anastomosis by means of transhepatic sharp recanalization was performed.

## Materials and Methods

Between May 2007 and November 2021, four patients (men, 75%; median 57 years, range 43–66 years) were referred for percutaneous biliary neo-anastomoses at a tertiary care center including liver transplant.

History, etiology, indication as well as procedural details for percutaneous biliary neo-anastomoses are reported in Table 1**.**

**Table 1:** Patient characteristics and technical issues

Patient (age/sex)	1 (41/M)	2 (64/M)	3 (66/F)	4 (50/M)
History	HCC due to chronic hepatitis B	HCC due to alcoholic liver cirrhosis	Adenocarcinoma of the pancreatic head	Klatskin tumor, type IIIb
Type of surgery	Living-related right hemiliver transplant	Living-related right hemiliver transplant	Robotically assisted Whipple procedure	Left hemihepatectomy and resection of segment I
Indication for neo-anasomosis	Exclusion of the right posterior hepatic duct	Bile leakage with biloma due to excluded right posterior hepatic duct	Exclusion of the right posterior hepatic duct	Leakage of the right posterior hepatic duct
Type of neo-anastomosis	BES	BBS	BES	BES
Biliary access	Puncture with 22G Chiba needle	Indwelling PTBD (8.5F)	Indwelling PTBD (8.5F)	Indwelling PTBD (8.5F)
Perforation device	Backend of a 0.018″ guidewire	Backend of a 0.018″ guidewire	Backend of a 0.018″ guidewire	Outback® catheter
Balloon size for neostomy dilatation	4 mm	2 mm/4 mm	4 mm	2 mm/4 mm
Initial PTBD size	8.5F	8.5F	8.5F	10F
Indwelling time of PTBD (days)	43	51	103	64
Upsizing	10.2F	10.2F	12F	None
Further neo-anastomosis balloon dilations	7-mm cutting balloon	None	None	5 mm
Adverse events	None	None	None	None
Follow-up time	Alive and well at 17 yrs	Death 7 weeks post BBS, due to sepsis	Alive and well at 31 months	Death 3 yrs post BES due to recurrent cancer

*M* male, *F* female, *HCC* hepatocellular carcinoma, *BES* bilio-entero-stomy, *BBS* bilio-bilio-stomy, *PTBD* percutaneous transhepatic biliary drainage

The indication for percutaneous biliary neo-anastomosis was made by a multidisciplinary board involving hepato-biliary-pancreatic surgeons, gastroenterologists and interventional radiologists.

Two of the four patients with leaking posterior right ducts were recipients of living-donor right hemi-liver transplants, which had been performed for hepatocellular carcinoma in cirrhotic livers. In two patients, major resections for biliary and pancreatic cancers had been performed. Three patients presented with peritonitis related to free intraabdominal bile leakage, and one patient had developed an extrahepatic biloma with recurrent septicemia.

All procedures were performed under local anesthesia and moderate sedation. Pre-procedural cross-sectional imaging (both CT and MRI) was reviewed. Attention was paid to perihilar vascular structures (hepatic artery, portal vein) in proximity to the planned neo-anastomosis. The excluded leaking bile duct had been identified on pre-procedural cross-sectional imaging, at fluoroscopy of indwelling biloma drains or percutaneous biliary drains (PTBD) placed via any other duct.

**Fig. 1:**
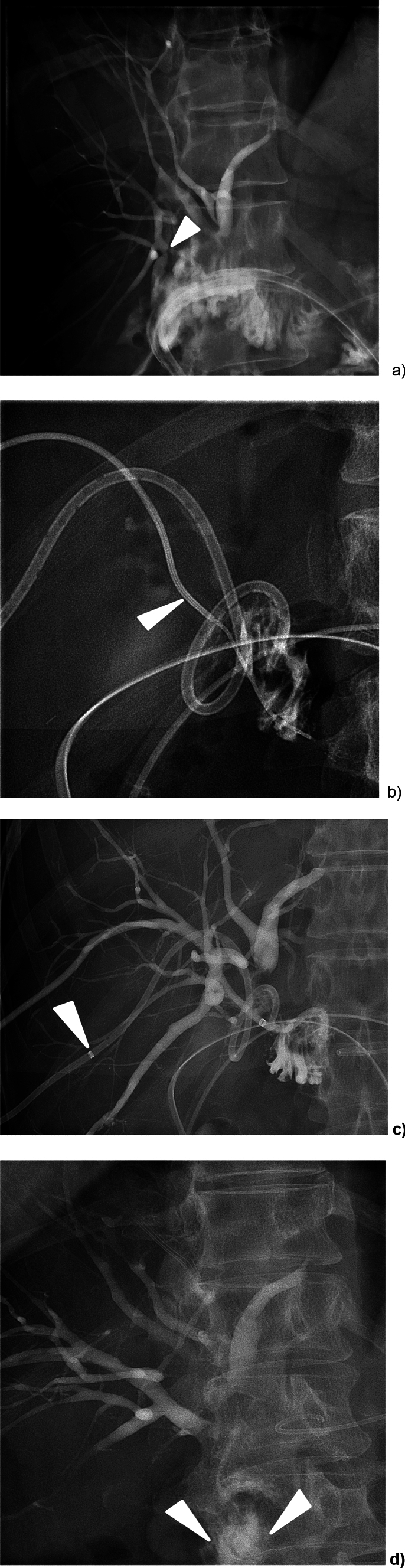
A 66-year-old female with adenocarcinoma of the pancreatic head (patient 3). Twelve days after Whipple’s procedure she presented with fever. **a** Transhepatic cholangiography shows a leak from the excluded right posterior sectoral duct (arrowhead). **b** A PTBD was placed via the right anterior ducts to opacify the small bowel loop to be accessed. A 8F sheath and a 4F Kumpe catheter were percutaneously advanced through the excluded posterior right duct. Penetration of the small bowel loop was achieved with the backend of a 0.018″ guidewire (arrowhead). **c** The newly created bilio-enteric anastomosis was secured with an 8.5F PTBD (arrowhead). **d** At removal of the PTBD, approximately 3 months after the intervention, there is proper flow of contrast agent through the bilio-entero-neo-anastomosis into the small bowel (arrowheads*)* without bile leakage

**Fig. 2:**
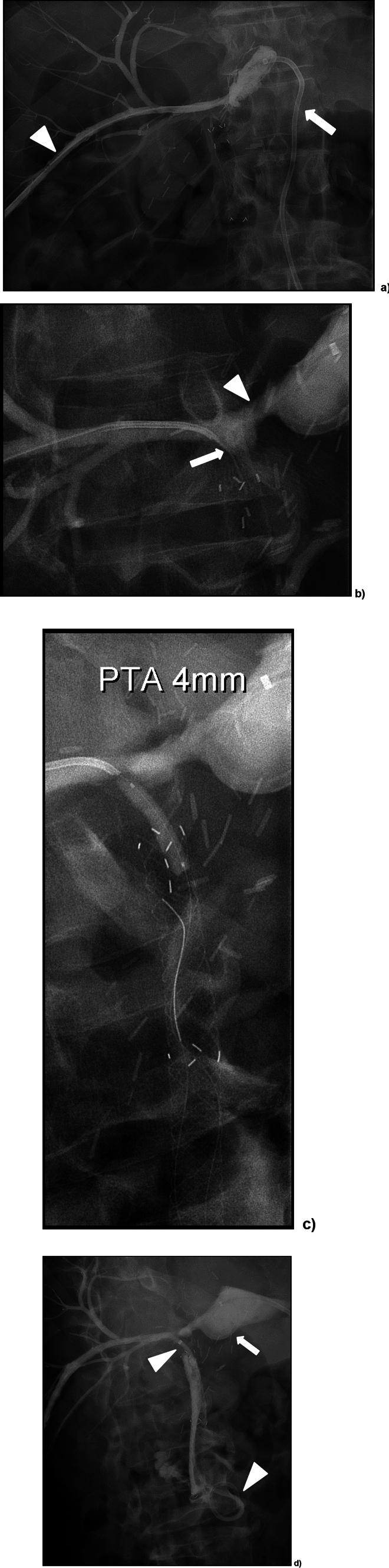
A 64-year-old male (patient 2) who had undergone living-donor right hemi-liver transplant because of HCC and cirrhosis presented with persisting fever at 6 weeks. **a** A biloma was noted next to the liver resection margin and percutaneously drained (arrow). Retrograde opacification of the posterior right duct, but no communication with the endoscopically placed biliary stent-graft was found as the biloma drain was injected. That excluded duct was percutaneously accessed (arrowhead). **b** The indwelling stent-graft in the recipients common hepatic duct was utilized as a target at the puncture with a bent reversed 0.018″ guidewire *(*arrow). There is a rather large bile duct defect (arrowhead) filling the biloma. **c** Balloon dilatation (4 mm) of the neo-anastomosis. **d** The newly created bilio-bilio-neostomy was secured with an 8.5F PTBD *(*arrowheads*).* The biloma drain was left in place *(*arrow*)*

Two techniques were utilized to perform the percutaneous biliary neo-anastomosis (PBNA). Both techniques aimed at creating a new connection between an excluded posterior right duct and an intestinal loop (*n* = 3, Fig. 1) or the common hepatic duct (*n* = 1, Fig. 2). Technical success was defined as an established connection without any leakage.

By means of a 22G Chiba needle the excluded posterior right hepatic duct was percutaneously transhepatically accessed. Vascular sheaths (8F, *n* = 3 or 10F, *n* = 1) were then introduced. A 5F catheter with an angled tip was then advanced as far as possible in the duct, followed by 2.7F coaxial microcatheter placement. Opacification of the bowel loop to be anastomosed was obtained in two cases by contrast injection via the PTBD. In the patient with the bilio-bilio-neo-anastomosis, the common hepatic duct (CHD) was easily visible under fluoroscopy due a previously placed stent-graft in the CHD. However, that stent-graft placed by endoscopy had failed to resolve the bile leak. In one patient the puncture of the bowel loop to be anastomosed was guided uniquely by fluoroscopy after review of the pre-procedural cross-sectional imaging depicting the anatomy of the adjacent small bowel.

In 3 out of 4 of cases, the stiff back end of a commonly available 0.018″ guidewire (V-18™, Boston Scientific Corp.) was used to access the enteric loop or the CHD. In order to direct this “sharp” recanalization, the tip of the reversed guidewire was bent accordingly. In one case, an Outback® Elite Re-entry catheter (Cordis, Miami Lakes FL, USA) was utilized. A microcatheter was advanced over the 018″ or, respectively, the 0.014″ guidewire through the presumed anastomosis. The correct enteric (*n* = 3) or CHD (*n* = 1) access was confirmed by means of contrast instillation. Dilatation of the track (2–4-mm-diameter angioplasty balloon) was then performed. Finally, the neo-anastomoses were secured by PTBDs (10.2F, *n* = 3; 12F, *n* = 1; Fig. 1).

The follow-up consisted of repeated cholangiographies via the PTBDs placed across the new anastomosis.

## Results

Technical success rate was 100%, as all four patients demonstrated patency of the neo-anastomosis and no bile leak.

The PTBDs were removed after a median of 65 days (range 43–103 days). The median follow-up was 2.8 years (range 0.13–17.0 years).

Two patients died 7 weeks and 3 years after PBNA of overwhelming sepsis or tumor recurrence. The first presented with recurrent bloody discharge from the PTBD 6 weeks after the neo-anastomosis. Computed tomography depicted a pseudoaneurysm of the right hepatic artery, which was treated with stent-grafting. Due to the time course and the anatomical localization of the pseudoaneurysm, its etiology most likely was the prior bile leakage or a perioperative lesion and not the percutaneous biliary neo-anastomosis per se. Hence, it was not deemed to be procedure-related. The second patient presented with cholangitis 20 months after the creation of the neo-anastomosis. Imaging showed a stenosis of the posterior right hepatic duct due to local tumor recurrence. The subsequently placed 8.5F PTBD remained in place until the death of the patient 16 months later.

## Discussion

The experience regarding iatrogenic bile duct injuries and their treatment has been described previously [5, 6]*.* However, in these two single- or multicenter series including a large number of patients the bile duct was injured at laparoscopic cholecystectomy. The 30-day morbidity and mortality rates of the repair by hepaticojejunostomy were 26% and 2%, respectively [6].

In the single-center study [5] just 36 out of 800 patients (4.5%) had Strasberg Type C injuries such as the four cases reported herein. Among all injuries, the interventional radiological contribution consisted of PTBDs (7.2%) or percutaneous abdominal drainages (28.2%). In addition, a recent review of the interventional radiological role in the treatment of Strasberg Type C injuries mentioned just hepaticojejunostomy (“preferred”) and PTBD (“sometimes”) as treatment options [7].

This small case series supports the concept that PBNA is technically feasible to treat Strasberg Type C injuries in patients who had prior complex HPB surgery including split-liver transplantation. These patients likely benefit from PBNA as an alternative to a redo bilio-jejunostomy. Due to the operative alteration endoscopic stenting of transected right posterior sectoral ducts may not be feasible.

Once the leaking posterior right sectoral duct has been percutaneously accessed, but the target (jejunum or CHD) puncture for a neo-anastomosis fails, any combination of bile duct embolization and ablative sclerosis may still be performed as a bail-out procedure [8].

Our experience in one case suggests that after pre-interventional review of hepatic CT and/or MR imaging, additional enteric target opacification may not be necessary.

A question that remains unsolved is the ideal point in time for PTBD removal. In our experience, PTBDs should probably be left in place for at least 6 weeks to create a new anastomosis.

This report has limitations related to the small number of patients affected by this operative complication, including the variety of indications and type of HPB surgery. However, all patients had symptomatic Strasberg Type C transections during complex HPB surgery and their repair in common.

## Conclusion

In conclusion, percutaneous biliary neo-anastomosis is technically feasible and safe to treat Strasberg C bile duct lesions after major HPB surgery using standard, readily available guidewires and catheters. The healing of these neo-anastomoses seems not to be dependent upon whether performed between a donor liver and its recipient, or after HPB surgeries in non-transplanted patients. Long-term post-interventional observation proves that these neo-anastomoses are durable.

## Abbreviations

PBNA: Percutaneous biliary neo-anastomosis
PTBD: Percutaneous biliary drainage
CHD: Common hepatic duct
HPB: Hepato-pancreato-biliary surgery
